# Reduced expression of the murine HLA-G homolog Qa-2 is associated with malignancy, epithelial-mesenchymal transition and stemness in breast cancer cells

**DOI:** 10.1038/s41598-017-06528-x

**Published:** 2017-07-24

**Authors:** Istéfani L. da Silva, Lucía Montero-Montero, Ester Martín-Villar, Jorge Martin-Pérez, Bruno Sainz, Jaime Renart, Renata Toscano Simões, Émerson Soares Veloso, Cláudia Salviano Teixeira, Mônica C. de Oliveira, Enio Ferreira, Miguel Quintanilla

**Affiliations:** 1 Department of General Pathology, Laboratory of Compared Pathology, Biological Science Institute, Federal University of Minas Gerais, 486, 31270-901 Belo Horizonte, Minas Gerais Brazil; 2 CAPES Foundation, Ministry of Education of Brazil, Brasilia, DF 70.040-020 Brazil; 3 Instituto de Investigaciones Biomédicas “Alberto Sols”, – Consejo Superior de Investigaciones Científicas (CSIC) - Universidad Autónoma de Madrid (UAM), 28029 Madrid, Spain; 4 Departamento de Biotecnología, Facultad de Ciencias Biosanitarias, Universidad Francisco de Vitoria, 28223 Madrid, Spain; 5 Enfermedades Crónicas y Cáncer Area, Instituto Ramón y Cajal de Investigación Sanitaria (IRYCIS), Madrid, Spain; 6 Institute of Education and Research of Santa Casa of Belo Horizonte, 590, 30150-240 Belo Horizonte, Minas Gerais Brazil; 7 Laboratory of Pharmacotecniques and Pharmaceutical Technologies, Pharmacy Faculty, Federal University of Minas Gerais, 486, 31270-901 Belo Horizonte, Minas Gerais Brazil

## Abstract

Qa-2 is believed to mediate a protective immune response against cancer; however, little is known about the role of Qa-2 in tumorigenesis. Here, we used 4T1 breast cancer cells to study the involvement of Qa-2 in tumor progression in a syngeneic host. Qa-2 expression was reduced during *in vivo* tumor growth and in cell lines derived from 4T1-induced tumors. Tumor-derived cells elicited an epithelial-mesenchymal transition associated with upregulation of Zeb1 and Twist1/2 and enhanced tumor initiating and invasive capacities. Furthermore, these cells showed increased stem characteristics, as demonstrated by upregulation of Hes1, Sox2 and Oct3/4, and enrichment of CD44^high^/CD24^median/low^ cells. Remarkably, Qa-2 cell-surface expression was excluded from the CD44^high^/CD24^median/low^ subpopulation. Tumor-derived cells showed increased Src activity, and treatment of these cells with the Src kinase inhibitor PP2 enhanced Qa-2 but reduced Sox2 and CD44^high^/CD24^median/low^ expression levels, suggesting that Src signaling, while positively associated with stemness, negatively regulates Qa-2 expression in breast cancer. Finally, overexpression of the Qa-2 family member Q7 on the cell surface slowed down *in vivo* tumor growth and reduced the metastatic potential of 4T1 cells. These results suggest an anti-malignant role for Qa-2 in breast cancer development, which appears to be absent from cancer stem cells.

## Introduction

HLA-G belongs to the human non-classical major histocompatibility complex (MHC), or MHC class 1b, that has been shown to be involved in the immune recognition of tumors^1, 2^. The genes encoding MHC class 1b antigens are oligomorphic, which grants an advantage with respect to the highly polymorphic MHC class 1a antigens in order to develop cancer immunotherapies directed to a wider patient population^3^. In this respect, it is important to understand the role MHC class 1b proteins play in cancer development and progression. Qa-2 is believed to be the murine homolog of HLA-G, as both families of proteins share a number of characteristics, including: *i*, the presence of membrane-bound and soluble forms that arise from alternative splicing; *ii*, their involvement in preimplantation embryo development; and *iii*, their immunoregulatory roles^2^. There are four main Qa-2 loci: *Q6*, *Q7*, *Q8* and *Q9*, which are present in different combinations in each mouse haplotype. The *Q7* gene is almost identical to *Q9*, and *Q6* is very similar to *Q8*. Therefore, these genes are referred as *Q7/Q9* and *Q6/Q8* pairs^4^.

It has been found that HLA-G expression is enhanced in a number of tumors, including different types of lymphomas and leukemias, melanoma, and breast, kidney, ovarian, lung and colorectal carcinomas^5^. Moreover, HLA-G expression is considered a bad prognostic factor in different types of solid tumors, including colorectal and breast cancers^5–7^. Whereas most studies have linked HLA-G expression with tumor immune evasion due to its interaction with inhibitory receptors on immune cells^5, 8–10^, other reports suggest that HLA-G can activate NK cells and promote cytotoxicity because of its interaction with the KIR2DL4 receptor^11, 12^. However, these results are controversial as both inhibitory and stimulatory functions have been reported for KIR2DL4, and it is unclear that HLA-G binds KIR2DL4 on NK cells in the tumor microenvironment^2, 5^. To date, however, only a handful studies have addressed the role of Qa-2 in cancer, and most of these studies have focused on Q9. Q9 expression is downregulated in cell lines derived from tumors, such as melanoma, hepatoma, mastocytoma and lymphoma^13, 14^, and has been involved in tumor rejection of melanoma, Lewis lung carcinoma and T-cell lymphoma^14–16^.

In this report, we used a 4T1 murine mammary carcinoma syngeneic model to analyze the expression of Qa-2 during *in vivo* breast cancer cell growth and in tumor cells lines derived from these tumors. 4T1 cells are a useful model for advanced human breast cancer or highly metastatic triple-negative carcinomas^17–19^. The role of Q7 in 4T1 tumor formation and metastasis was also assessed. Our results suggest an anti-tumor function for Qa-2 in breast cancer.

## Results

### Qa-2 expression levels decrease during tumor formation

**Figure 1:**
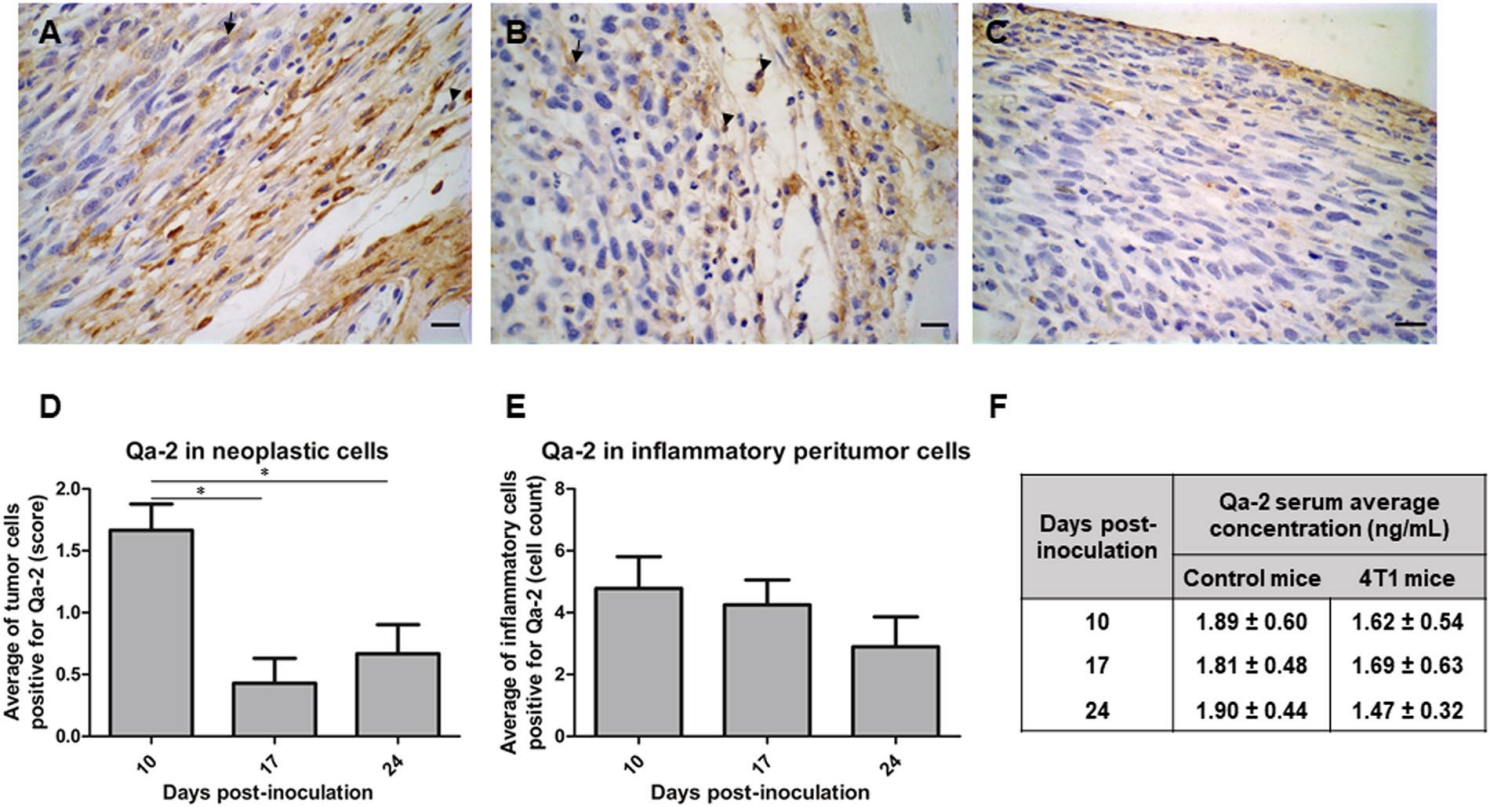
Qa-2 expression decreases during tumor growth. (**A**–**C**) Immunohistochemical detection of Qa-2 in 4T1-induced tumors at 10 (**A**), 17 (**B**) and 24 (**C**) days post-inoculation. Examples of stained tumor cells and peritumor inflammatory cells are indicated by arrows and arrowheads, respectively. Groups of mice (n = 15) were inoculated i.d./s.c. with 10^6^ cells into the left flank. Bars, 20 μm. (**D**,**E**) Estimation of neoplastic (**D**) and peritumor inflammatory (**E**) cells stained for Qa-2. Values represent the average number of tumor cells stained for Qa-2 determined in 15 fields. A number of 5 tumors per each post-injection time were evaluated. Asterisk indicates statistically significant difference (^*^ *p* < 0.05). (**F**) The concentration of soluble Qa-2 in the sera of mice bearing 4T1 tumors and control mice without tumors at different days post-injection, as determined by ELISA. Values represent the average of 5 mice per group at each post-inoculation time.

In order to evaluate whether Qa-2 expression changes during breast cancer development, 4T1 cells were intradermally (i.d.)/subcutaneously (s.c.) injected into the left flank of syngeneic Balb/c mice and tumors harvested at 10, 17 and 24 days post-injection. At these post-injection times, the mean volumes of tumors were 1.47 ± 0.75, 1.93 ± 0.68 and 4.61 ± 1.66 cm^3^, respectively. Qa-2 expression in neoplastic and peritumor inflammatory cells was determined by immunohistochemistry, whereas soluble Qa-2 concentrations in the sera of the animals were scored by ELISA. The presence of Qa-2 in tumors was focal (Fig. 1A–C). The number of neoplastic cells that stained positive for Qa-2 was, in general, low, and never exceeded 25% of the total number of tumor cells. Moreover, a clear observable and significant decrease in Qa-2 expression in neoplastic cells was associated with tumor expansion (Fig. 1D). The number of peritumor inflammatory Qa-2-positive cells and the amount of soluble Qa-2 were also reduced during tumor growth; however, these differences were not statistically significant (Fig. 1E,F).

### Transplantation *in vivo* selects for 4T1 cells with enhanced fibroblastic, malignant and stem characteristics

**Figure 2:**
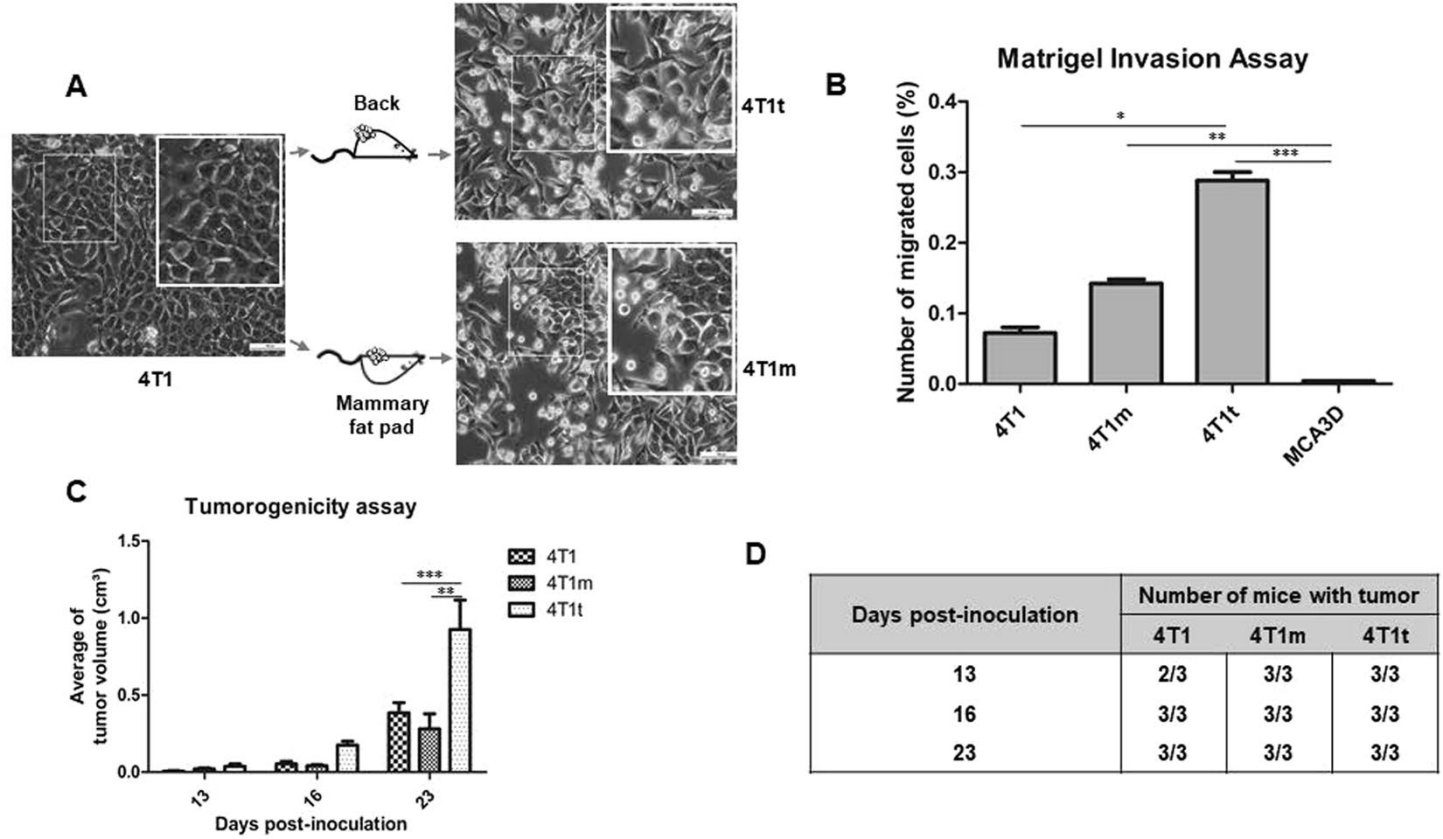
Cell lines derived from 4T1-induced tumors are more malignant. (**A**) Origin and phase contrast micrographs of 4T1t and 4T1m cell lines. Bars, 100 μm. Insets show increased magnifications of the indicated areas. (**B**) Matrigel invasion assay. Cells were seeded on a 24-well Matrigel-coated invasion chamber. FBS (5%) was used as a chemoattractant. Migrated cells on the underside of the filter were fixed, stained and counted. Values are the percentage of migrated cells with respect to the total cells seeded on the well, and represent the means of triplicate assays from three independent experiments. Asterisks indicate statistically significant difference (^*^ *p* < 0.05; ^**^ *p* < 0.01; ^***^ *p* < 0.001) (**C**) Tumorigenic behavior of 4T1-derived cell lines. Groups of mice (n = 3) were inoculated i.d./s.c. with 10^3^ cells into the left flank. The size of tumors was measured at the indicated times with a caliper and the volume calculated as indicated in Materials and Methods. Asterisks indicate statistically significant difference (^**^ *p* < 0.01; ^***^ *p* < 0.001). (**D**) The incidence of tumors at different indicated post-injection times.

Primary tumors were induced by injection of 4T1 cells either i.d./s.c. into the back or, orthotopically, into the mammary fat pad of Balb/c mice. Compared to 4T1 parental cells, tumor-derived cells, particularly 4T1t cells, showed a more elongated morphology and a higher number of detached (stringent) cells, as determined by microscopic examination (Fig. 2A, insets). While 4T1t cells were less proliferative than 4T1m and 4T1 cells *in vitro* (Supplementary Fig. S1), they showed increased invasive characteristics (Fig. 2B) and secreted higher amounts of MMP-9 metalloproteinase (Supplementary Fig. S2). 4T1m cells, on the other hand, exhibited a similar proliferative capacity (Supplementary Fig. S1), but were more invasive than parental 4T1 cells (Fig. 2B). A non-invasive MCA3D mouse keratinocyte cell line was included in these assays as a control. 4T1t and 4T1m cells were also more tumorigenic than 4T1 upon re-injection into mice. Since 4T1 cells are already strongly tumorigenic, only 10^3^ cells were inoculated per site into recipient animals in order to compare the tumor initiating capacity of each cell line. All mice inoculated with 4T1t and 4T1m cells developed tumors of 0.021–0.063 cm^3^ at 13 days post-injection, whereas only 2 out of 3 mice inoculated with 4T1 cells harbored tumors of smaller volumes (Fig. 2C,D). Interestingly, tumors induced by 4T1t cells grew more rapidly than those induced by 4T1 and 4T1m cells (Fig. 2C), in light of its lower proliferative capacity *in vitro*.

**Figure 3:**
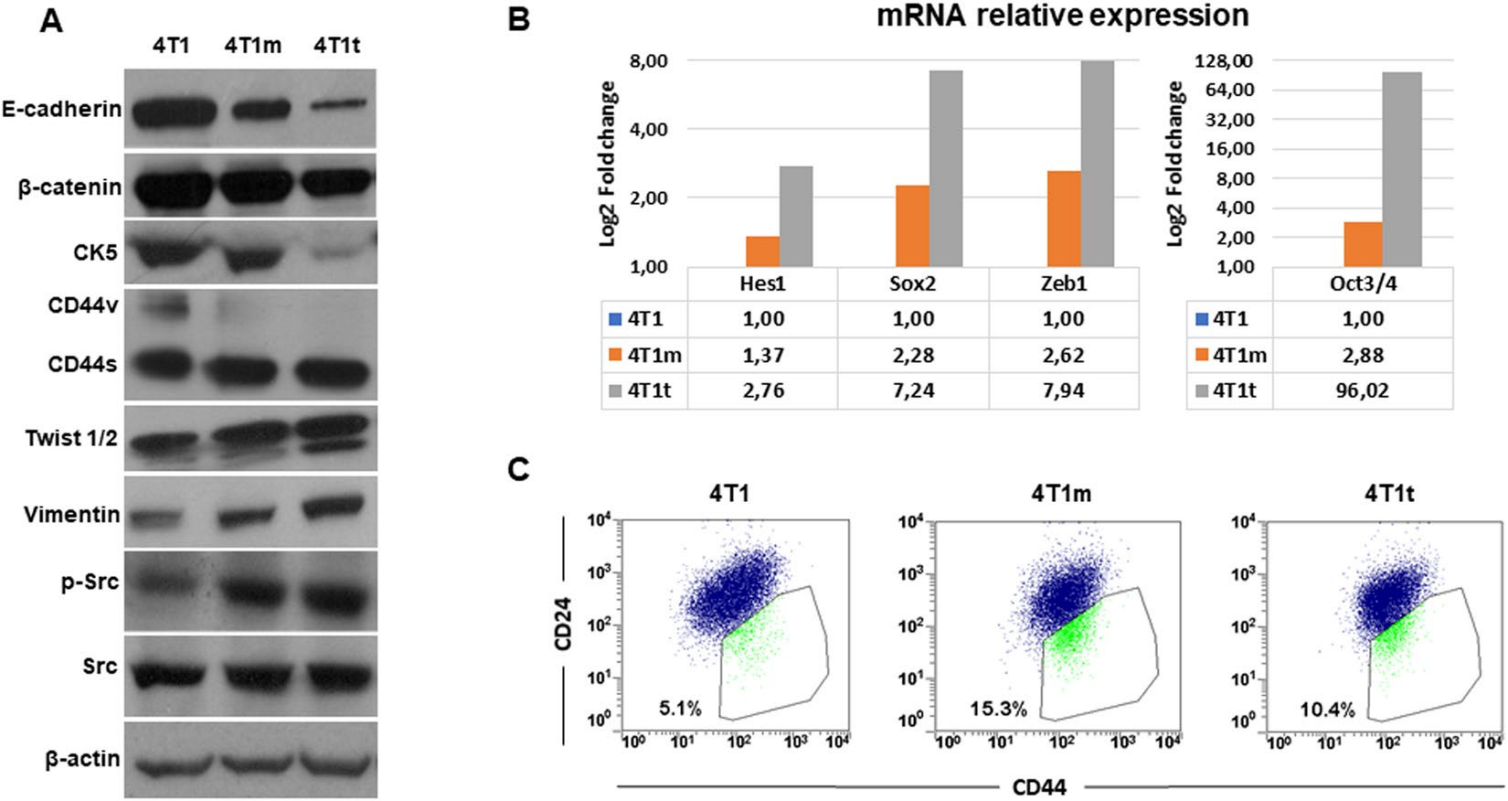
4T1 cells transplanted *in vivo* undergo a partial EMT and acquire stem-like cell characteristics. (**A**) Western blot analysis of EMT protein markers and Src. Active Src (p-Src) was detected with a mAb specifically recognizing Src phosphorylated on Y418. β-actin was used as a control for protein loading. The blot shown is representative of two independent experiments. (**B**) Analysis of mRNA expression of the indicated transcription factors. Normalized transcript levels were measured by real-time qRT-PCR relative to hprt mRNA levels. Values (mean of duplicate determinations) represent increases with respect to 4T1 parental cells, which were given an arbitrary value of 1. (**C**) Flow cytometry analysis of the cell-surface markers CD44 and CD24 in the indicated cell lines. The percentage of CD44^high^/CD24^med/low^ cell subpopulation is indicated. A representative experiment out of two is shown.

Since 4T1m and 4T1t cells showed a more fibroblastic appearance compared to 4T1 cells, we assessed the expression of differentiation protein markers by Western blotting. Both 4T1m and 4T1t cells showed reduction of epithelial markers, such as E-cadherin, β-catenin and cytokeratin 5 (CK5). Conversely, these cells showed increased expression of vimentin, a mesenchymal marker (Fig. 3A). A loss of variant isoforms of the hyaluronan receptor CD44 (CD44v) and increased expression of the standard isoform, CD44s, was also observed in these cells (Fig. 3A). This shift in CD44 expression has also been related to EMT^20, 21^. A quantification of these changes by densitometric analysis is shown in Supplementary Fig. S3. Interestingly, the observed changes in differentiation markers correlated with enhanced expression of the transcription factors Twist 1/2 (Fig. 3A) and Zeb1 (Fig. 3B), two master regulators of EMT^22^, and with enhanced activity/autophosphorylation of Src while the total levels of Src were unchanged (Fig. 3A).

Since EMT has been associated with the generation of stem-like characteristics^23, 24^, we also analyzed the expression of stemness-related factors by real-time qRT-PCR. As shown in Fig. 3B, the expression of Hes1, Sox2 and Oct3/4, three transcription factors involved in the maintenance and self-renewal of stem cells^25–27^, was increased in both 4T1m and 4T1t cell lines (particularly in the latter) with respect to 4T1 parental cells. In addition, we analyzed the expression of CD44 and CD24 by flow cytometry. These two cell-surface markers have been associated with breast cancer stem cells (CSCs), specifically the CD44^high^/CD24^median/low^ subpopulation^23^. CD44^high^/CD24^med/low^ were enhanced 2–3 fold in 4T1m and 4T1t cells with respect to the parental cell line (Fig. 3C), indicating enrichment in CSC-like cells.

Overall, these results suggested that 4T1-induced tumors select cells that undergo an EMT associated with increased tumorigenic and stem characteristics and activation of the cytoplasmic tyrosine kinase Src.

### Transplantation *in vivo* of 4T1 cells reduces Qa-2 expression

**Figure 4:**
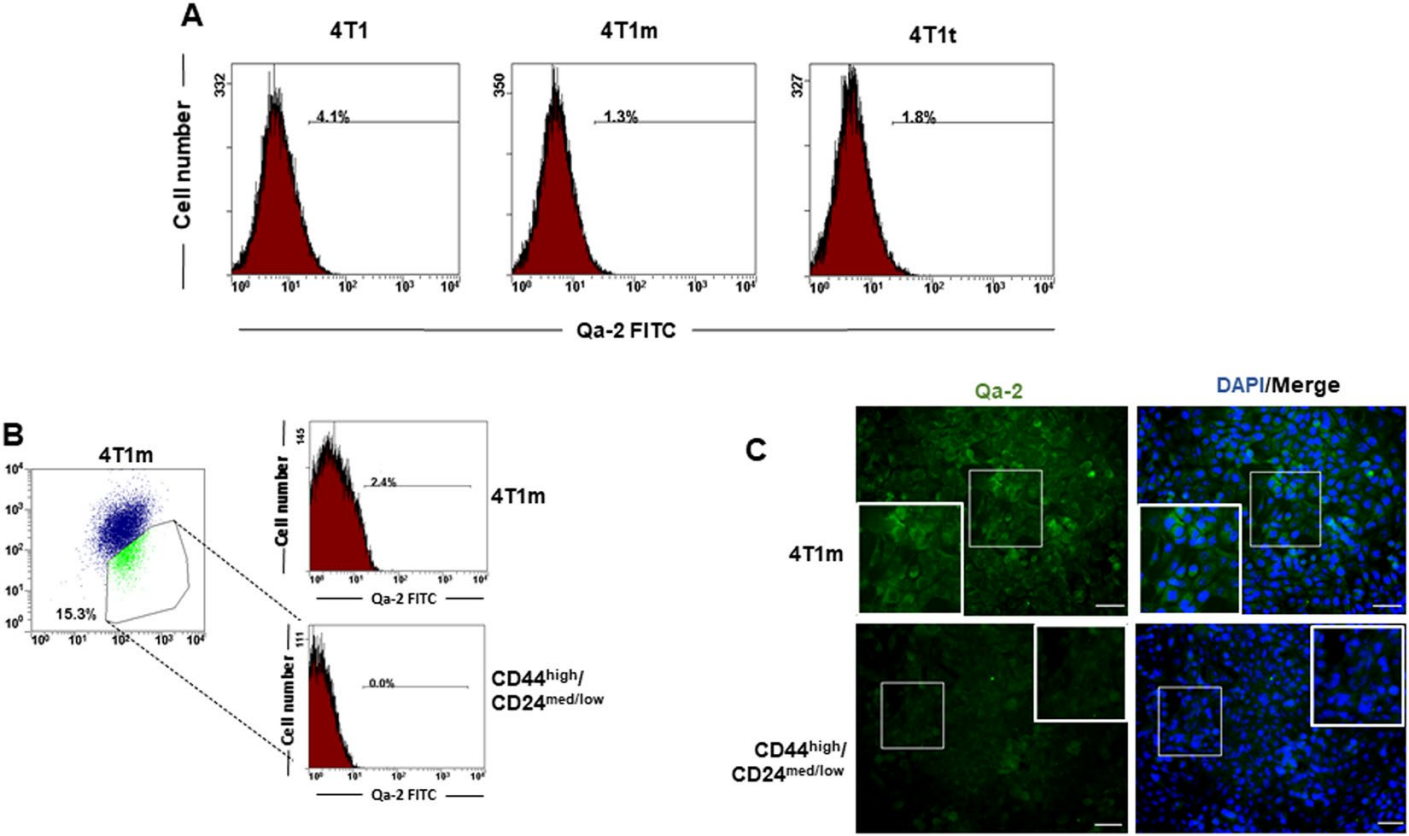
Cell lines derived from 4T1 tumors have reduced Qa-2 expression. (**A**) Analysis of Qa-2 cell-surface expression by flow cytometry. (**B**) The CD44^high^/CD24^med/low^ cell subpopulation was isolated from 4T1m cells by FACS and the expression of Qa-2 determined in these cells (bottom panel) and in the total 4T1m cell population (upper panel). Note that Qa-2 expression in CD44^high^/CD24^med/low^ cells is undetectable. (**C**) Immunofluorescence detection of Qa-2 in 4T1m and CD44^high^/CD24^med/low^ cells. Nuclei were stained with DAPI. Bars, 100 μm. Insets show increased magnifications of the indicated areas.

The expression of Qa-2 was also analyzed in 4T1 and 4T1-derived cell lines by flow cytometry. We found that cell-surface Qa-2 expression was reduced approximately 2–3 fold in 4T1m and 4T1t cells with respect to 4T1 cells (Fig. 4A). In order to analyze the relationship of Qa-2 with stemness, we isolated the CD44^high^/CD24^med/low^ cell population from 4T1m cells by fluorescence-activated cell sorting (FACS) and assessed the presence of Qa-2 in these cells. As shown in Fig. 4B, Qa-2 expression in CD44^high^/CD24^med/low^ cells was negligible. This finding was confirmed by immunofluorescence analysis (Fig. 4C), suggesting an inverse relationship between Qa-2 expression and stem-like cells in breast cancer. In addition, the results obtained in this cell model indicate an inverse correlation between Qa-2 levels and tumor progression.

### Inhibition of Src stimulates Qa-2 expression

**Figure 5:**
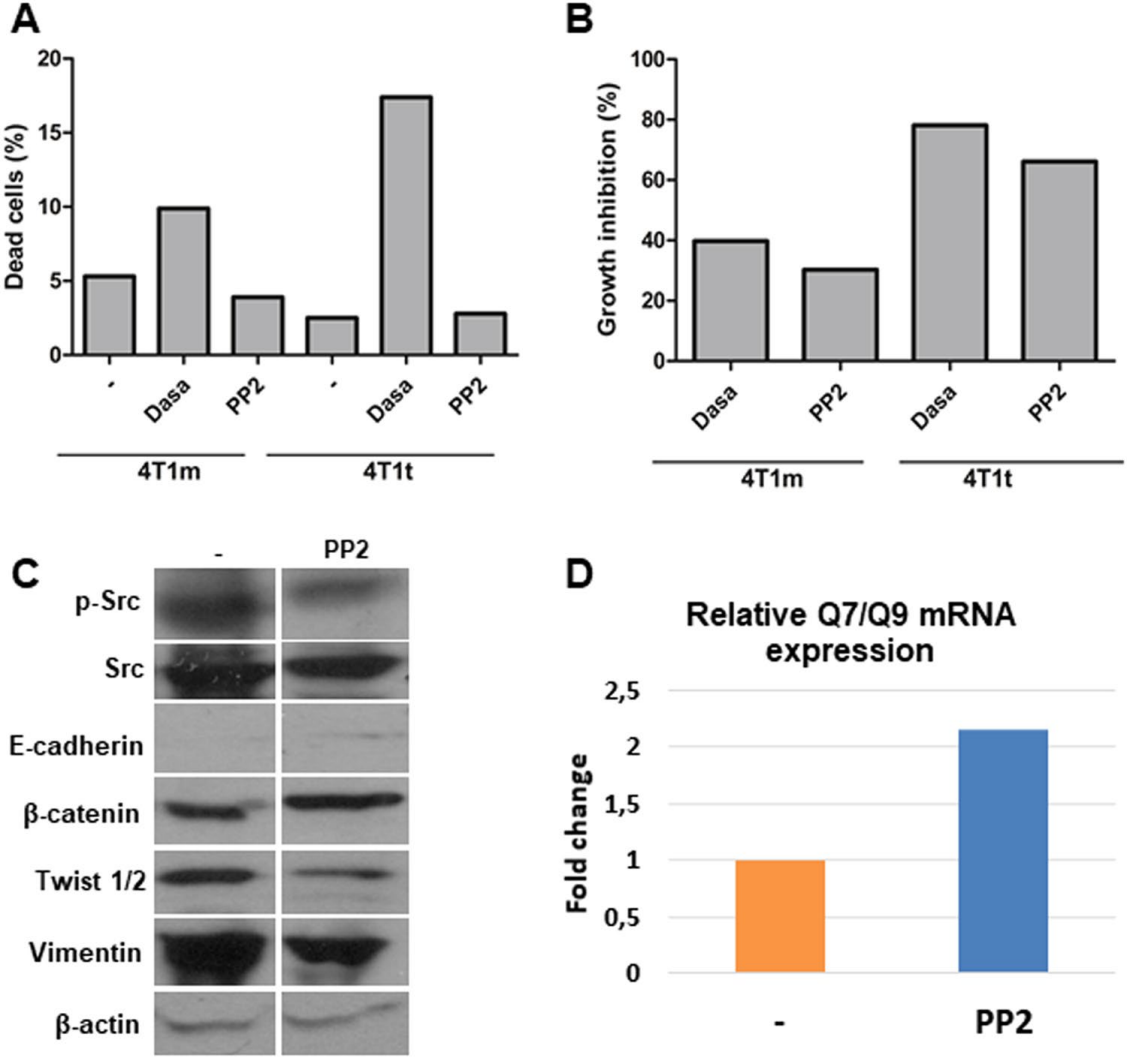
Pharmacological inhibition of Src enhances Qa-2 expression. (**A**) Effect of Src inhibitors on cell viability. 4T1t and 4T1m cell cultures were treated with Dasatinib (Dasa; 100 nM) and PP2 (5 μM) for 48 h, and cell viability was determined by Trypan blue exclusion. Values represent the means of duplicate incubations. (**B**) Effect of Src inhibitors on cell growth. Cultures were treated as specified in panel A, and cells were counted at the end of the experiment. (**C**) Effect of PP2 (5 μM) on the expression of Src and EMT protein markers of 4T1t cells. Protein levels were determined by Western blot analysis. β-actin was used as a control for protein loading. (**D**) Analysis of Q7/Q9 mRNA expression. Transcript levels were measured by real-time qRT-PCR, normalized to β-actin mRNA levels, and values for PP2-treated cells were compared to untreated cells, which were given an arbitrary value of 1.

**Figure 6:**
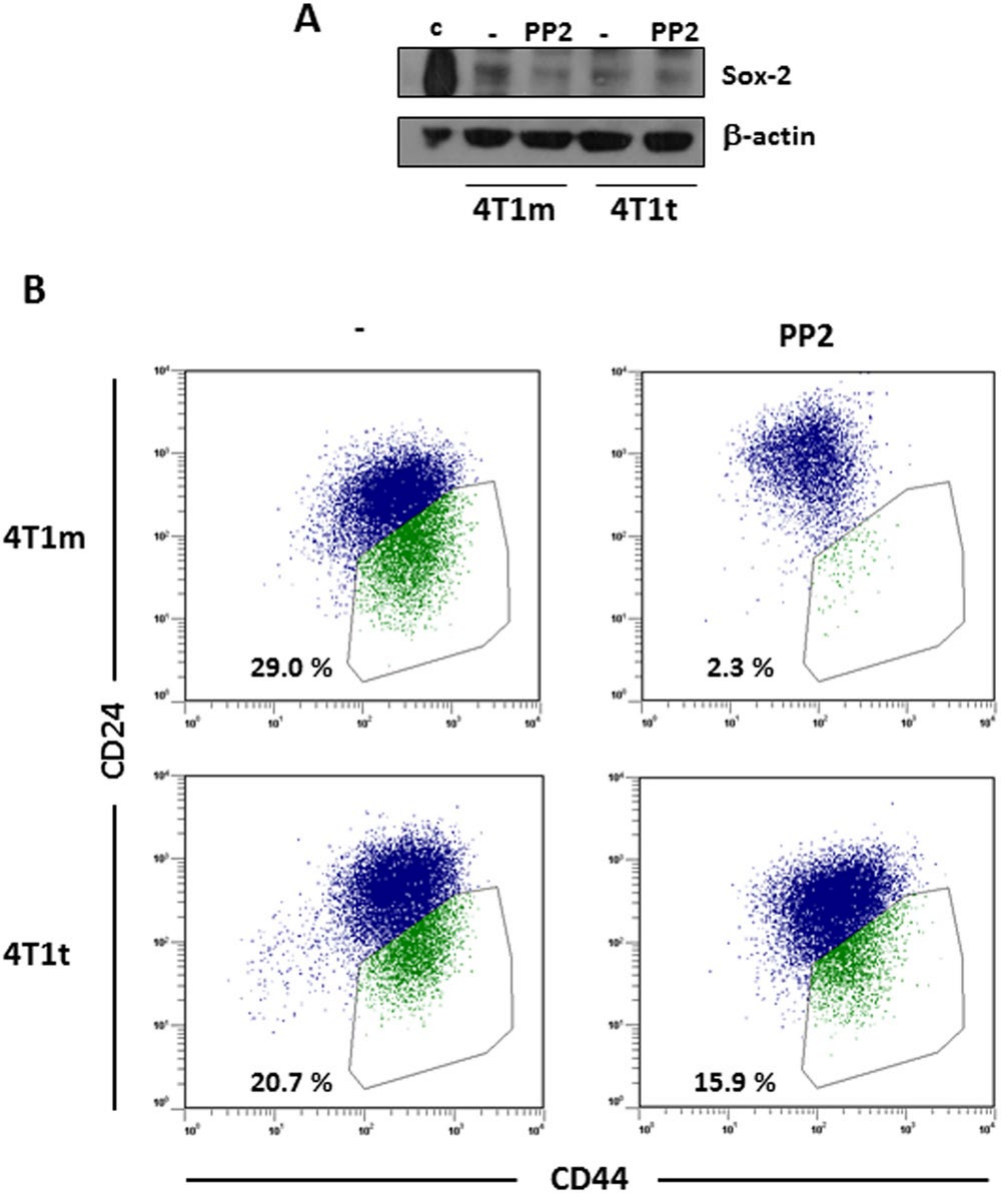
Pharmacological inhibition of Src reduces stemness. (**A**) Western blot analysis of Sox2 expression in 4T1m and 4T1t cells untreated or treated with PP2. Due to the high background observed in the blot, a positive control with a lysate of 293 T cells overexpressing Sox2 (c) was included. β-actin was used as a control for protein loading. (**B**) Flow cytometry analysis of the cell-surface markers CD44 and CD24 in 4T1m and 4T1t cells untreated or treated with PP2. The percentage of CD44^high^/CD24^med/low^ cell subpopulation is indicated. A representative experiment out of two is shown.

Since the Src family kinases (SFKs) has been involved in EMT^28, 29^ and pluripotency^30^, and Qa-2 appears to mediate activation of the SFK member Fyn^31^, we treated 4T1m and 4T1t cell lines with two well-established inhibitors of SFK activity: Dasatinib and PP2^32^. Dasatinib at a concentration of 100 nM had an apparent cytotoxic effect on the cultures, causing a high proportion of cell death in both cell lines. In contrast, PP2 at 5 μM was more benign inducing much lower cell death (Fig. 5A and Supplementary Fig. S4), and promoted a reduction in the number of stringent cells, particularly in 4T1t cells (Supplementary Fig. S4). Both inhibitors reduced 4T1t and 4T1m cell growth, although the effect on 4T1t cells was stronger (Fig. 5B). PP2 efficiently reduced Src activity in 4T1t cells, as shown by decreased levels of phospho-Src. This effect occurred concomitantly with a slight increase in the synthesis of E-cadherin and β-catenin and a reduction of vimentin and Twist1/2 protein levels (Fig. 5C and Supplementary Fig. S5). Similar results were obtained in 4T1m cells treated with the kinase inhibitor (Supplementary Fig. S6). PP2 also induced a clear increase of about 2 fold in the levels of Qa-2, as assessed by real-time qRT-PCR determination of Q7/Q9 transcripts, two members of the Qa-2 family (Fig. 5D). In addition, we analyzed the effect of PP2 on stemness-related factors by Western blotting. While antibodies used for Hes1 and Oct3/4 did not recognize any specific protein in the cell lysates, we found that PP2 induced a decrease on the levels of Sox2, more evident in 4T1m cells (Fig. 6A). Moreover, a reduction in the CD44^high^/CD24^med/low^ cell subpopulation was also observed in 4T1t and 4T1m cell lines after treatment with PP2 (Fig. 6B).

### Qa-2 overexpression induces a less aggressive tumor phenotype

**Figure 7:**
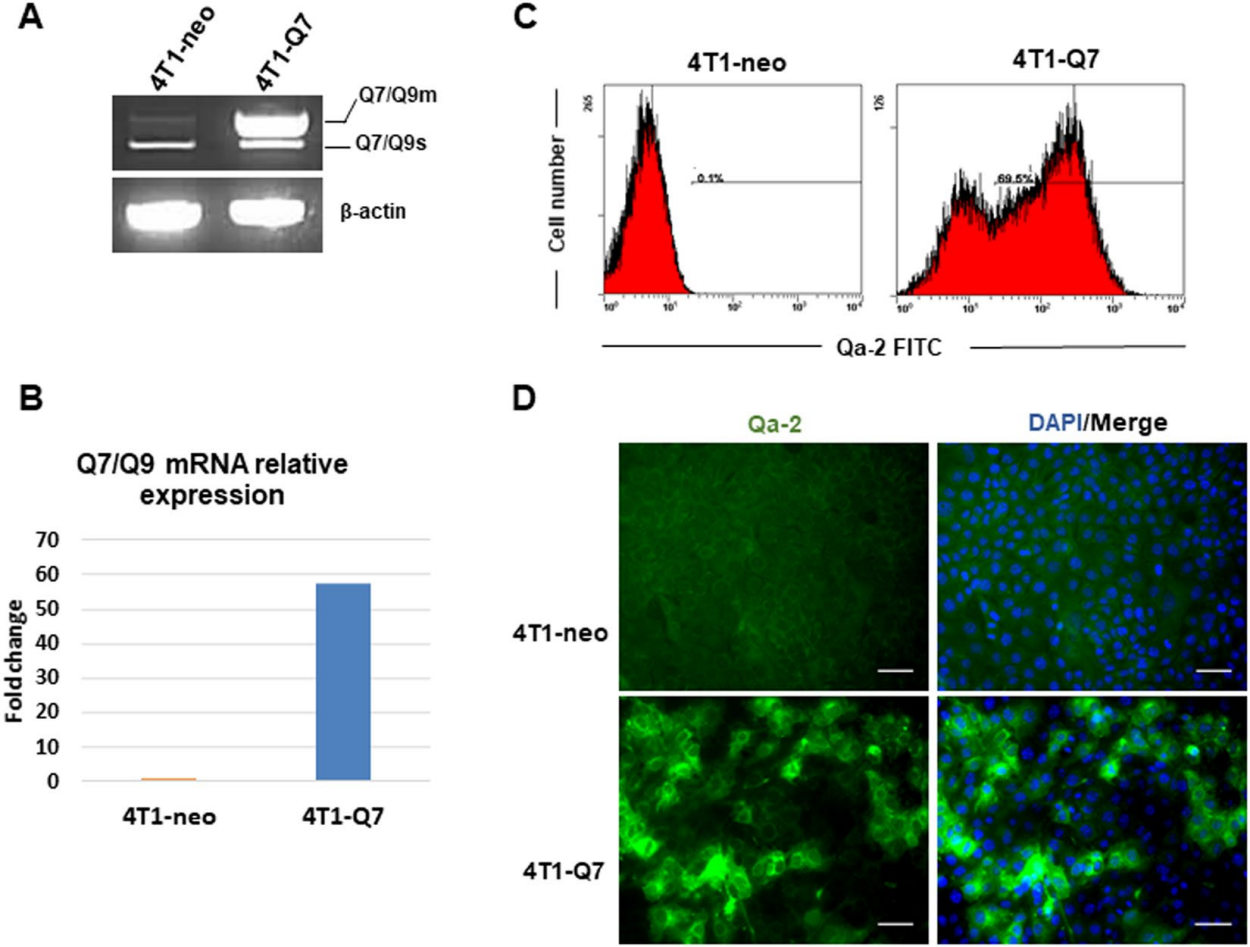
Stable transfection of Q7 cDNA into 4T1 cells. (**A**) Semiquantitative RT-PCR analysis of Q7 expression. Q7/Q9m, membrane-associated form; Q7/Q9s, soluble form. (**B**) Analysis of Q7 mRNA expression. Transcript levels were measured by real-time qRT-PCR, normalized to β-actin mRNA levels, and values for 4T1-Q7 were compared to 4T1-neo, which were given an arbitrary value of 1. (**C**) Analysis of Q7 cell-surface expression by flow cytometry. (**D**) Immunofluorescence detection of Q7. Nuclei were stained with DAPI. Bars, 100 μm.

In order to evaluate the effect of Qa-2 modulation on tumor growth and metastasis *in vivo*, 4T1 cells-expressing luciferase were stably transfected with Q7 cDNA cloned in the pcDNA3 expression vector. Enhanced expression of Q7 in 4T1-Q7 transfectants with respect to control 4T1-neo cells (transfected with the vector alone) was confirmed by semi-quantitative RT-PCR (Fig. 7A), real-time qRT-PCR (Fig. 7B), flow cytometry (Fig. 7C) and immunofluorescence (Fig. 7D) analysis. Overexpression of Q7 in 4T1 cells did not induce a significant morphological change or variations in the expression of differentiation protein markers, as determined by Western blotting (data not shown).

**Figure 8:**
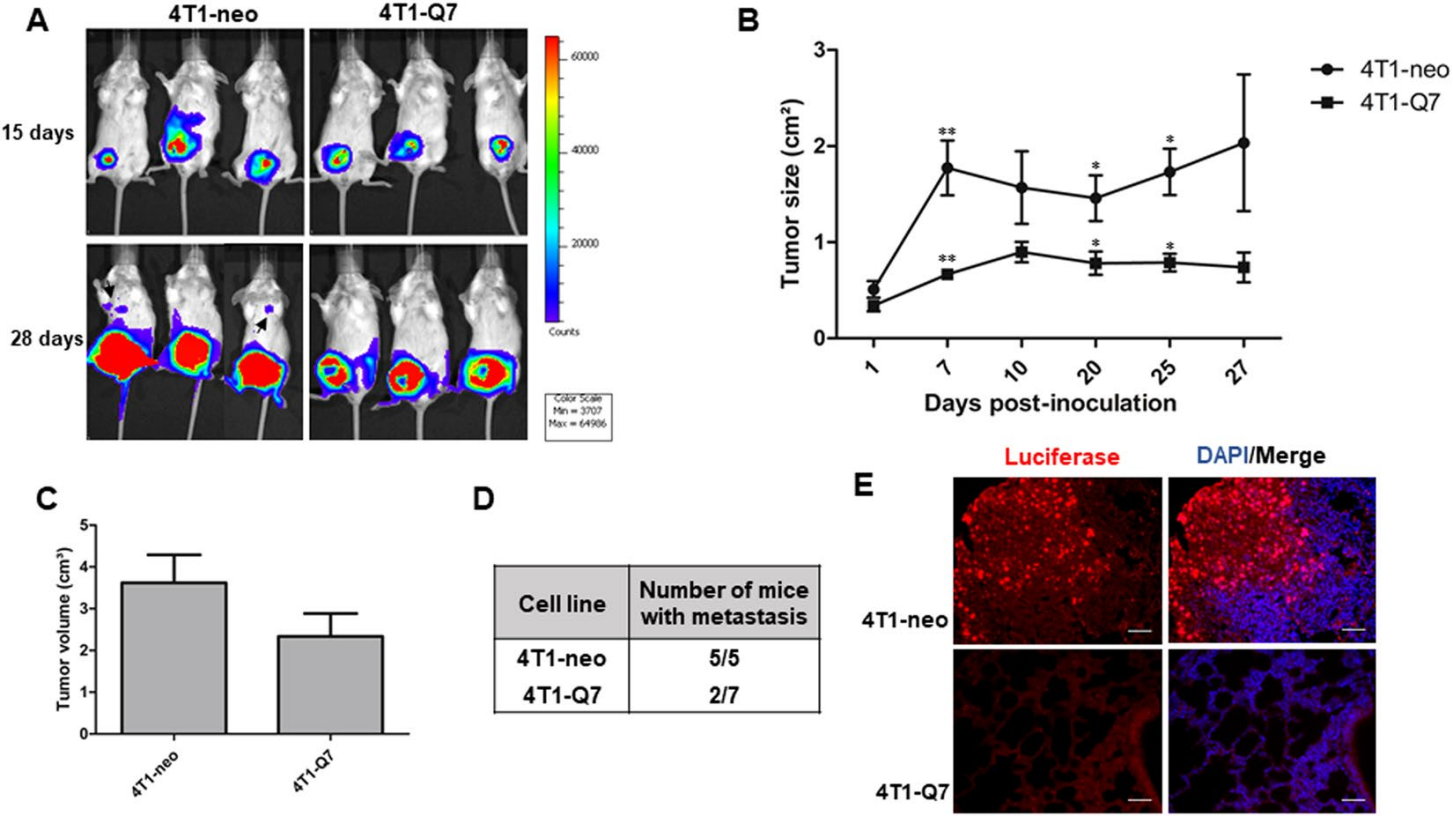
Overexpression of Q7 reduces tumor growth and metastatic potential of 4T1 cells. (**A**) Representative whole body bioluminescence images of mice with tumors induced by the indicated cell lines at 15 and 28 days post-injection. Note that 4T1-neo, but not 4T1-Q7 mice show metastatic colonies at 28 days (arrows). (**B**) Tumor growth was monitored at different post-injection days by measuring the bioluminescence area as indicated in Materials and methods. Asterisks indicates statistically significant differences (^*^ *p* < 0.05; ^**^ *p* < 0.01). (**C**) Measurement of the volumes of primary tumors at 28 days post-inoculation. n.s., statistically non-significant. (**D**) Incidence of pulmonary metastases at 28 days post-inoculation. (**E**) Immunofluorescence detection of luciferase in lungs of mice with mammary tumors induced by 4T1-neo and 4T1-Q7 cells at 28 days post-injection. Nuclei were stained with DAPI. Bars, 100 μm.

4T1-Q7 and 4T1-neo cells were orthotopically injected into the mammary fat pad of Balb/c mice and tumor growth monitored at different times by *in vivo* imaging. 4T1-Q7 cells induced tumors that grew slower than those induced by 4T1-neo cells (Fig. 8A,B). At the end of the experiment (28 days post-injection), tumor volumes (determined by caliper measurements) induced by 4T1-Q7 were appreciably less than those induced by control 4T1-neo cells, although differences were not statistically significant (Fig. 8C). Strikingly, 4T1-Q7 cells were less metastatic than control 4T1-neo (Fig. 8A, arrows) or 4T1 parental (data not shown) cells. In fact, only about 30% of mice inoculated with 4T1-Q7 cells developed lung metastases *versus* 100% of 4T1-neo mice (Fig. 8D). The presence of metastatic cells in the lungs was confirmed by luciferase immunostaining of histological sections. Shown in Fig. 8E is a representative image of a lung section from a mouse injected with 4T1-neo cells, where a large number of metastatic tumor cells can be observed, whereas no metastatic tumor cells can be seen in a lung section from a mouse injected with 4T1-Q7 cells.

## Discussion

In this study, we show that Qa-2 protein levels diminish during the growth of tumors produced *in vivo* by highly tumorigenic 4T1 breast cancer cells in syngeneic mice. Furthermore, cell lines established from 4T1-induced tumors (either in the back or in the mammary fat pad) showed a marked reduction in Qa-2 cell-surface expression. These cell lines, 4T1t and 4T1m, had stronger tumor initiating and invasive capacities compared to 4T1 cells, and elicited an EMT associated with upregulation of EMT-promoting transcription factors; i.e., Twist1/2 and Zeb1^22^, as well as factors related to stemness, such as Hes1, Sox2 and Oct3/4^25–27^. These results are in agreement with previous reports suggesting that EMT generates cells that acquire malignant and stem-like traits^23, 24^. Likewise, we isolated a CD44^high^/CD24^med/low^ cell population from 4T1m cells, which are enriched in breast CSCs^23, 33, 34^, in order to study the potential link between Qa-2 and CSCs. We found that CD44^high^/CD24^med/low^ cells do not express Qa-2. To our knowledge, this is the first time that exclusion of the MHC class 1b proteins from CSCs is reported. The above results suggested an anti-tumor role for Qa-2. This assumption was confirmed by forcing the expression of Q7, a key member of the Qa-2 family in Balb/c mice^13, 35^, on the surface of 4T1 cells by cDNA transfection. 4T1-Q7 cells induced tumors that grew slower and produced less lung metastases than control or parental cells.

The anti-oncogenic role of Qa-2 is in deep contrast with the reported pro-tumorigenic function for HLA-G^2, 5^ (see also the Introduction), which raises the question whether Qa-2 is the functional murine homolog of HLA-G, at least in the context of cancer. Our results are in line with studies reported by Chiang and Stroynowski on the protective role exerted by Q9 for melanoma, lung carcinoma and T-cell lymphoma *in vivo* outgrowth. In these studies, restoration of Q9 expression in tumors that have downregulated Q9 results in a CD8^+^ cytotoxic T lymphocytes (CTL)-mediated response that eliminates them in syngeneic hosts^14–16^. In other report, the same authors have shown that Q9 expressed in class I-deficient melanoma cells protects cells from NK cell-mediated cytolysis^36^. In this respect, Qa-2 exclusion from CD44^high^/CD24^med/low^ cells might be related to immune evasion of this breast CSC-like cell subpopulation, a hypothesis that remains to be investigated. Indeed, when mice were challenged with 4T1 cells overexpressing Q7 on their surface tumors that developed grew at a slower rate and were less aggressive than those induced by control cells, which might suggest that Q7 renders tumors susceptible to immune surveillance; hence, downregulation of Qa-2 during *in vivo* 4T1 tumor growth. Alternatively, an immune-independent mechanism might be behind the anti-tumorigenic effect of Q7, as 100% of mice challenged with 4T1-Q7 cells developed tumors as did mice challenged with control 4T1-neo cells. Further work is needed to clarify this issue.

We also provide evidence in this study that Src signaling negatively regulates Qa-2 expression in breast cancer cells, as inhibition of Src activity by the well-established PP2 inhibitor enhanced Qa-2 transcript levels. Interestingly, patients with triple negative breast cancer appear to be sensitive to treatment with pharmacological inhibitors of Src^37^. The PP2-mediated increase in Qa-2 expression levels occurred concomitantly with a reduction in Sox2 expression and the number of CD44^high^/CD24^med/low^ cells, emphasizing again the inverse relationship between Qa-2 and stemness in breast cancer cells. Also, PP2 induced a slight reversion of EMT, as shown by decreased expression of Twist1/2 and vimentin and an observable increase in E-cadherin and β-catenin levels. These results suggest at least a partial role for Src activity in the enrichment of CSC cells and EMT induced by transplantation of 4T1 cells *in vivo*. Augmented Src activity has been found in several types of malignancies, including breast cancer, associated not only with increased tumor cell proliferation, but also with EMT, invasion and metastasis^28, 29^.

In summary, we show in this report an inverse relationship between Qa-2 expression and malignancy, epithelial-mesenchymal transition and stemness in breast cancer that appears to be partially mediated by Src signaling. However, more studies are necessary to unveil the mechanism(s) behind these connections, and to decipher the exact role of Qa-2 in malignancy.

## Materials and Methods

### Cell culture and treatments

The 4T1 cell line was obtained from the American Type Culture Collection (ATCC, USA). 4T1m and 4T1t cell lines were derived by explanting tumors induced by 4T1 cells in the mammary fat pad (see below) and the back (i.d./s.c.) of Balb/cj mice, respectively. Tumors had a size 0.5–1 cm-diameter when explanted. MCA3D are non-tumorigenic keratinocytes derived from mouse skin treated with a chemical carcinogen^38^. Cell lines were routinely cultured in Dulbecco’s modified Eagle’s medium (DMEM), except MCA3D cells that were grown in Ham’s F-12 medium, supplemented with 1% penicillin/streptomycin and 10% fetal bovine serum (FBS; Gibco), at 37 °C, in a humidified 5% CO_2_ atmosphere. All cell lines were tested for mycoplasma contamination by immunofluorescence staining with 1 μg/ml solution of 4′,6-diamino-2-phenilindole (DAPI; Sigma–Aldrich).

Cells were treated with Src family kinase inhibitors Dasatinib (100 nM) and PP2 (5 μM), as previously described^32^. Briefly, ~ 6 × 10^5^ cells were seeded in duplicate plates, and incubated in the presence of inhibitors for 48 h. At the end of the experiment, cells were counted and the percentage of growth inhibition calculated with respect to vehicle-treated controls. Cell viability was determined by 0.4% Trypan blue exclusion.

### cDNA transfection

The H2-Q7 antigen full-length coding sequence was amplified by PCR using the following primers: forward: 5′-CAGTGTGCTGGAATTCATGGCTCTAACAATGCTGCTC-3′, and reverse: 5′-GATATCTGCAGAATTCCCACCTGTGTTTCACCTCCTA-3′, and the Phusion^®^ High Fidelity DNA polymerase (New England Biolabs). The PCR product was subcloned into the pcDNA3 expression vector (Invitrogen) with In-Fusion HD Cloning kit (Clontech). The presence and integrity of the insert was verified by DNA sequencing. 4T1 cells were transfected with this plasmid, or the pcDNA3 vector alone (control), using Lipofectamine 2000 (Invitrogen), and transfected cells selected in 0.5 mg/ml G418 (Invitrogen) for 3 weeks.

### Matrigel invasion assay

Invasion assays were performed using Transwell chambers with 8-μm-pore polycarbonate filters coated with Matrigel (BD Biosciences). Approximately 2 × 10^4^ cells were seeded in the upper compartment in medium without serum, and allowed to transmigrate for 24 h using 5% FBS as a chemoattractant in the bottom compartment. Non-migrated cells on the upper side of the Transwell were subsequently removed, and those on the underside were fixed with methanol and stained with 0.1% crystal violet. Cell migration was quantified by counting the number of cells that migrated through the filters. Ten different fields (x200) were counted in triplicate experiments.

### Quantitative and semiquantitative RT-PCR

RNA from cell lines was purified using the RNeasy kit (Qiagen). Quantitative reverse transcription-PCR analysis was performed using the high-capacity cDNA Reverse Transcription kit (Applied Biosystems) in a 7900HT Fast (Life Technologies) instrument. Taqman probes for Qa-2 (Mm00843895_s1) and β-actin (Mm00843895_s1), used as an internal control, were purchased from Life Technologies. Amplification of Zeb1, Sox2, Hes1 and Oct3/4 transcription factors was performed using Power SYBR^TM^ Green Master Mix (ThermoFisher), with hypoxhantine-guanine phosphoribosyl transferase (hprt) as an internal control. Oligonucleotide sequences for these primers are detailed in Supplementary Information.

For semiquantitative RT-PCR, RNAs (1 μg) were incubated with the Moloney murine leukemia virus reverse transcriptase (Promega), and the generated cDNA were used for PCR amplication. Specific primers for Q7/Q9 and β-actin amplification have been described elsewhere^13^.

### Western blot and ELISA analysis

For Western blot analysis, cells were lysed in RIPA buffer containing a cocktail of protease and phosphatase inhibitors, as described^39^. For immunodetection of proteins, the following primary antibodies (Abs) were used: mAb ECCD for E-cadherin (dilution 1:250), kindly provided by Dr A. Cano (Instituto de Investigaciones Biomédicas Alberto Sols, Spain), mAbs 610153 (1:2000) and RV202 (1:250) for β-catenin and vimentin, respectively, from BD Biosciences; mAb EPR1600Y (1:1500) for CK5 from Abcam; mAb 327 (1:500) for Src was a kind gift of Dr. J.S. Brugge (Harvard Medical School, USA) and polyclonal Ab recognizing Src phosphorylated Y418 (1:1000) was from Biosource Int. (Invitrogen); polyclonal Ab for Twist1/2 (1:1000) was from Genentech; mAb AC-74 (1:10000) for β-actin from Sigma-Aldrich; mAb KM-201 (1:200) for CD44 was a kind gift from Dr. H. Yarwood (Imperial College London, UK); and polyclonal goat IgG for Sox2 (AF2018, R&D Systems), a generous gift from Dr. I. Palmero (Instituto de Investigaciones Biomédicas Alberto Sols, Spain). Appropriate horseradish-peroxidase (HRP)-conjugated IgGs were used as secondary antibodies. Peroxidase activity was visualized using an enhanced chemiluminescence kit as indicated by the manufacturer (Pierce).

The concentration of Qa-2 in the sera of mice was determined using a Qa-2 ELISA kit (MyBiosource.com) following the manufacturer’s recommendations.

### Flow cytometry and sorting

The following antibodies were used for FACS analysis: fluorescein isothiocyanate (FITC)-conjugated anti-CD44 (clone KM201) and anti-Qa-2 (clone 69H1–9–9, eBioscience), and phycoerytrin (PE)-conjugated anti-CD24 (clone M1/69, Miltenyi Biotech), in a FACS Canto II with FACSDiva software (BD Bioscience) or in a Cytomics FC 500 MPL with MXP software (Beckman Coulter). Positivity for Qa-2 expression was identified using mouse IgG2a kappa isotype control (FITC) (Supplementary Fig. S7). As positive control, C57Bl/6 mouse splenocytes were used (Supplementary Fig. S7). CD44^high^ expression level was selected arbitrarily to include cells having fluorescence intensity units (FI) higher than 50. Similarly, CD24^low^ expression level was selected to include cells having FI lower than 50, and CD24^median^ expression level cells with FI between 50 and 400. Sorting of the CD44^high^/CD24^med/low^ cell subpopulation from 4T1m cells was performed using a FACSVantage SE sorter (BD Biosciences).

### Tumorigenicity and bioluminescence assays

All animal experiments were approved by the Animal Care and Use Committees of the Spanish National Research Council (CSIC), Community of Madrid (Ref. PROEX 37/14) and Federal University of Minas Gerais of Brazil. Mice were cared for following institutional guidelines and in accordance with the standards established by the European Union (2010/63/UE) and the National Institutes of Health Guide for the Care and Use of Laboratory Animals.

For tumorigenicity assays, 10^6^ or 10^3^ cells (as indicated) were i.d./s.c. injected into the left flank of 6 to 8-weeks old female Balb/cj mice (Harlan). The size of tumors was calculated via caliper measurements of two orthogonal diameters at different times. The tumor volume was calculated using the equation *V* = *a* × *b* ^2^/2, where *a* is the largest diameter and *b* is the smallest diameter. Tumors were fixed in formalin and embedded in paraffin.

For experiments to evaluate the tumorigenic and metastatic potential of 4T1-neo and 4T1-Q7 cell lines, mice were anesthetized with isofluorane inhalation before orthotopic transplantation of cells. An incision of ~ 0.5 cm was made along the medial side of the nipple of the second right mammary gland. Approximately, 10^5^ cells, stably infected with lentiviral expression particles for luciferase (CMV-Luciferase firefly, from AMSBIO, Abingdon, UK), were injected into the exposed fat pad, and the skin layer was subsequently closed with surgical staples. Each mouse was imaged periodically, using an IVIS Lumina 2 imaging system (Xenogen, USA), after retro-orbital injection of 3 mg luciferin (Goldbio, USA) in 100 μl of PBS under anesthesia. The areas of tumors (ROI, regions of interest) were determined at different post-inoculation times according to Living Image 4.0 software (Perkin Elmer). After 28 days, mice were euthanasized and lungs excised for immunofluorescence analysis. Pieces of lungs were fixed in formalin and embedded in paraffin.

### Immunohistochemical and immunofluorescence analysis

Immunohistochemical detection of Qa-2 was performed in deparaffinized tumor sections, after heat-induced antigen retrieval, by the Envision plus peroxidase method (Dako). Clone 69H1-9-9 (1:50) was used as the primary Ab for Qa-2 detection. The reaction product was developed with diaminobenzidine tetrahydrochloride and H_2_O_2_. The sections were dehydrated in graded ethanols, cleared in xylene, and mounted in Permont after counterstain with Hematoxylin. A total number of 15 sections (x60) were evaluated. Workshop score for Qa-2 expression in tumor cells was as follows: + 1 when up to 10% of cells were stained; +2 when 11–25% of cells were stained; and +3 when > 25% of cells were stained.

Immunofluorescence detection of Qa-2 in cultured cells was performed in confluent cultures grown on glass coverslips, washed in PBS and permeabilized with 0.05% Triton X-100, using FITC-conjugated anti-Qa-2 mAb (clone 69H1-9-9, 1:50; eBioscience). Coverslips were mounted on ProLong^®^ Diamond Antifade Mountant with DAPI (Thermo Fisher Scientific) and examined with a fluorescence microscope (Nikon Eclipse 90i).

For immunolocalization of luciferase in deparaffinized lung histological sections, a goat polyclonal Ab against luciferase (1:100; Novus Biologicals) was used after permeabilization in 0,1% Triton X-100 and heat-induced antigen retrieval in 1 mM EDTA. The primary Ab signal was amplified using donkey anti-goat IgG secondary Ab coupled to HRP and TSA Plus Cyanine (Perkin Elmer), following the manufacturer’s instructions. Cell nuclei were counterstained with DAPI.

### Statistics

The data are shown as the mean ± SEM. All data were normalized by Kolmogorov-Smirnov test and Grubbs’ test for outliers. Comparison between two groups was performed using the Student’s *t* test and for unpaired data by the Mann-Whitney U test. Significance was determined using the one-way analysis of variance (ANOVA) or the Student’s *t* test. Differences were considered significant if *P* < 0.05. All statistical analyses was performed using GraphPad Instat 5.0 software.

## Electronic supplementary material

Supplementary Information
