# Supplementary material for: Reduced expression of the murine HLA-G homolog Qa-2 is associated with malignancy, epithelial-mesenchymal transition and stemness in breast cancer cells
Source: Sci Rep. 2017 Jul 24;7:6276. doi: 10.1038/s41598-017-06528-x (PMC5524840; doi:10.1038/s41598-017-06528-x)
Supplement: Supplementary file 1 — Supplementary Information [file 41598_2017_6528_MOESM1_ESM.pdf]

SUPPLEMENTARY INFORMATION

Reduced expression of the murine HLA-G homolog Qa-2 is associated with malignancy, epithelial-mesenchymal transition and stemness in breast cancer cells.

Istéfani L. da Silva[1, 2, 3], Lucía Montero-Montero[3], Ester Martín-Villar[3], Jorge Martin-Pérez[3], Bruno Sainz Jr[3], Jaime Renart[3], Renata Toscano Simões[4], Émerson Soares Veloso[1], Cláudia Salviano Teixeira[5], Mônica C. de Oliveira[5], Enio Ferreira[1] & Miguel Quintanilla[3 *].

[1]Department of General Pathology, Laboratory of Compared Pathology, Biological Science Institute. Federal University of Minas Gerais, 486, 31270-901 Belo Horizonte, Minas Gerais, Brazil.

[2]CAPES Foundation, Ministry of Education of Brazil, Brasilia, DF 70.040-020, Brazil.

[3]Instituto de Investigaciones Biomédicas "Alberto Sols" – Consejo Superior de Investigaciones Científicas (CSIC) - Universidad Autónoma de Madrid (UAM), 28029-Madrid, Spain.

[4]Institute of Education and Research of Santa Casa of Belo Horizonte, 590, 30150-240, Belo Horizonte, Minas Gerais, Brazil

[5]Laboratory of Pharmacotecniques and Pharmaceutical Technologies, Pharmacy Faculty, Federal University of Minas Gerais, 486, 31270-901 Belo Horizonte, Minas Gerais, Brazil

* Corresponding author: M.Q. (email: mquintanilla@iib.uam.es)

Supplementary Methods

qRT-PCR

Specific primers for amplification of murine transcription factors and hprt using Power SYBR^{TM} Green Master Mix (ThermoFisher) were as follow:

Zeb1:

5’-GAGCCGCCAGTGAAGGTGATC-3’

5’-GTGAGGCCTCTTACCTGTGTGCT-3’

Sox2:

5’-TAGAGATAGACTCCGGGCGATGA-3’

5’-TTGCCTTAAACAAGACCACGAAA-3’

Hes1:

5’-TGCCAGCTGATATAATGGAGAA-3’

5’-CCATGATAGGCTTTGATGACTTT-3’

Oct3/4:

5’-TCTTTCCACCAGGCCCCCGGCTC-3’

5’-TGCGGGCGGACATGGGGAGATCC-3’

Hprt:

5’-TCCTCCTCAGACCGCTTTT-3’

5’-CCTGGTTCATCATCGCTAATC-3’

Proliferation assay

~ $5x10^3$ cells/well were seeded in a 96-well plate in complete medium in triplicate and grown for 24 h. Proliferation was evaluated using the XTT colorimetric cell proliferation assay kit (ATCC, USA) following the manufacturer’s recommendations. Absorbance at 475 nm and 660 nm was measured using a microplate reader, and the relative proliferation rate calculated by Specific Absorbance as follow: A475nm(Test) – A475nm(Blank) – A660nm(Test).

Zymography

Metalloproteinase (MMP) activity was determined in sera-free media conditioned by 48 h, and evaluated by SDS-PAGE zymograms containing 2% gelatin, as described elsewhere (1).

Isolation of murine splenocytes for Qa-2 flow cytometry analysis

The spleen of a C57Bl/6 mouse was cut into pieces and squeezed with forceps in PBS. Red blood cells were lysed with a buffer containing 8.3 g/L ammonium chloride in 0.01M Tris-HCL pH 7.4, and splenocytes rinsed twice in FACS buffer containing 0.5% BSA in PBS. Cells were filtered through a 100 μm mesh strainer, resuspended in FACS buffer, counted and diluted at the desired concentration.

References

1. Toth, M., Sohail, A. & Fridman, R. Assesment of gelatinases (MMP-2 and MMP-9) by gelatin zymography. *Methods Mol Biol* **878**, 121-135 (2008).

Supplementary Figure Legends

Figure S1. Cell proliferation assays. ~ $5x10^3$ cells/well were seeded in a 96-well plate in medium plus serum. After 24 h, cultures were pulsed with XTT for 7 h, and absorbance determined as indicated.

Figure S2. MMP9 activity secreted by the cell lines. Subconfluent cell cultures were conditioned for 48 h by serum-free medium, and gelatinase activity determined as indicated in Supplementary Methods. Values indicated at the bottom are expressed relative to 4T1 cells, in which an arbitrary value of 1 was given.

Figure S3. Quantification of EMT protein markers expression changes. The intensity of the bands of the Western blot showed in Fig. 3A were quantified by densitometric analysis, and values expressed relative to 4T1 cells, in which an arbitrary value of 1 was given.

Figure S4. Phase contrast micrographs of 4T1m and 4T1t cells before and after treatment with Src kinase inhibitors. Cells were treated with Dasa (100 nM), PP2 (5 μM), and vehicle for 48 h. Bars, 100 μm

Figure S5. Quantification of Src and EMT protein markers expression changes after 4T1t treatment with PP2. The intensity of the bands of the Western blot showed in Fig. 5C were quantified by densitometric analysis, and values expressed relative to untreated cells, in which an arbitrary value of 1 was given.

Figure S6. Effect of PP2 on the expression of EMT protein markers in 4T1m cells. Protein levels were determined by Western blot analysis. β-actin was used as a control for protein loading.

Figure S7. Isotype control for flow cytometry analysis of Qa-2 cell-surface expression. 4T1m cells and splenocytes were stained with IgG2a kappa isotype control and anti-Qa-2 antibody.

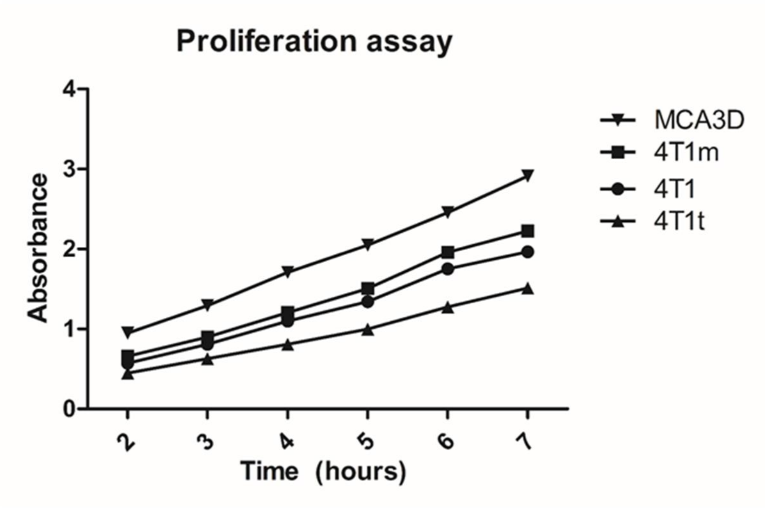

Figure S1

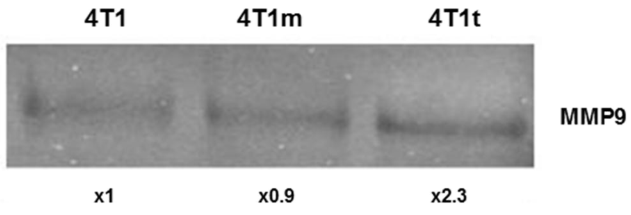

Figure S2

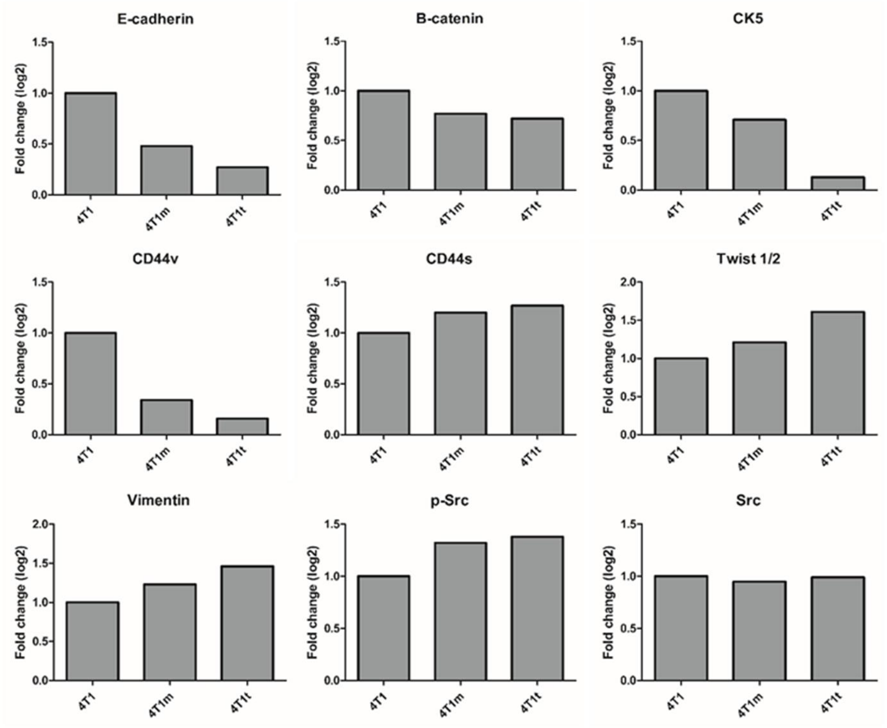

Figure S3

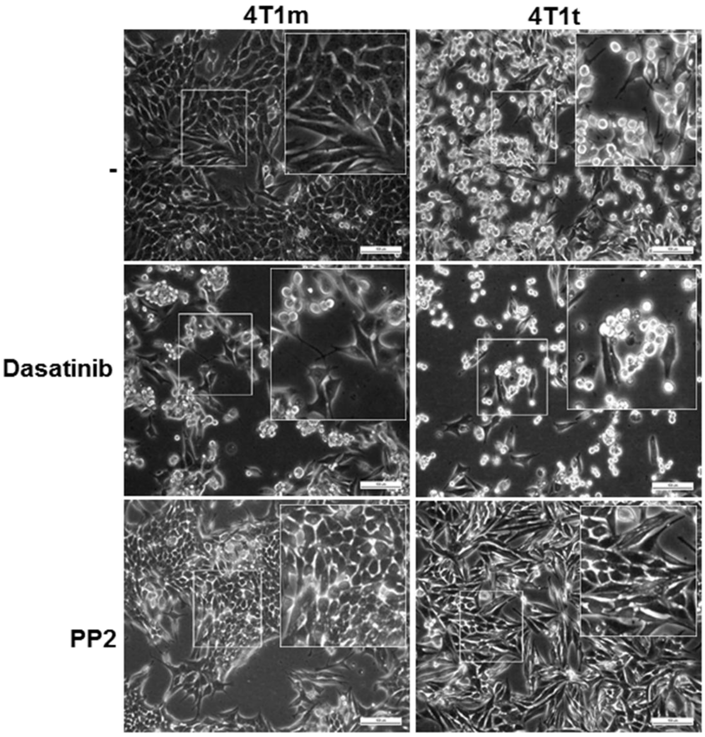

Figure S4

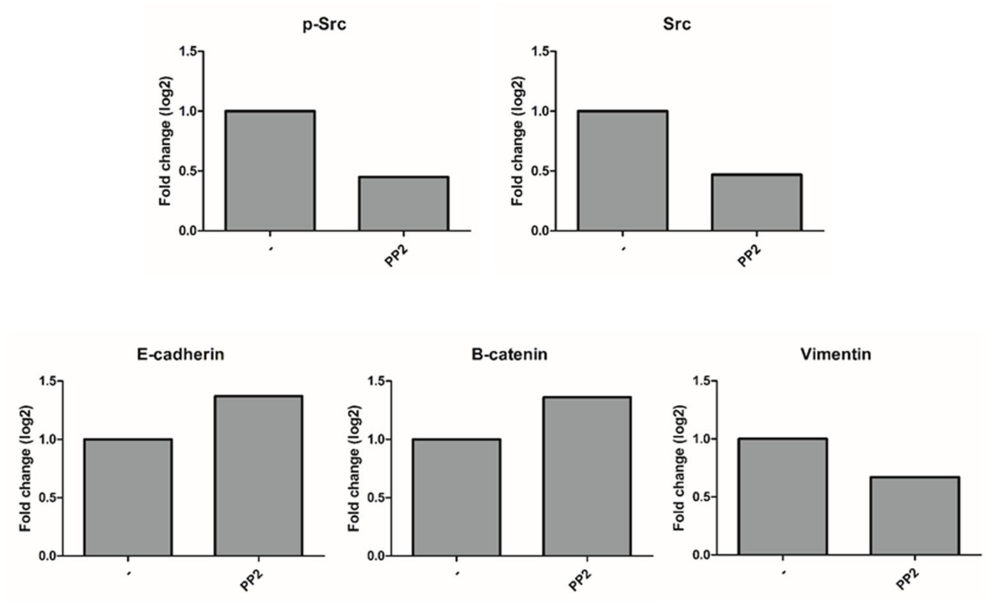

Figure S5

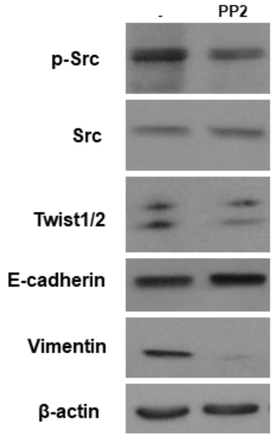

Figure S6

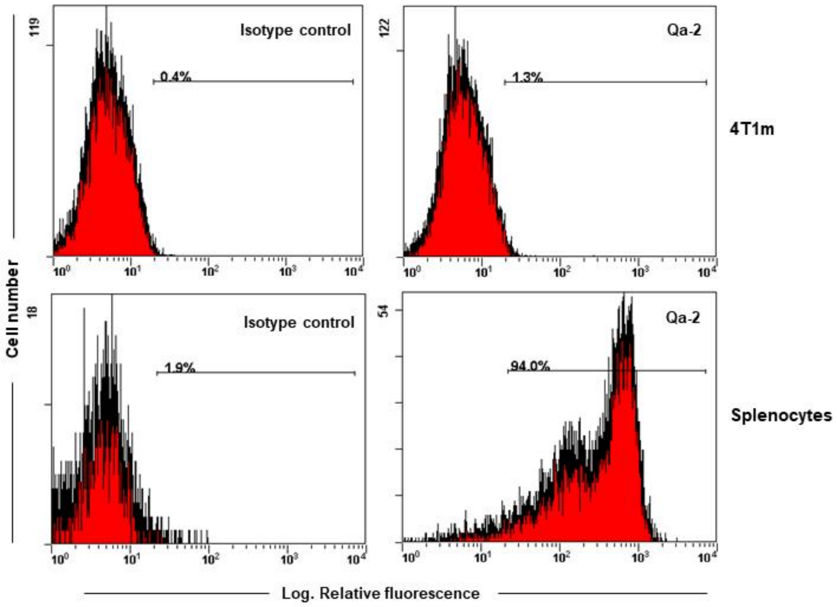
Isotype control
0.4%
Qa-2
1.3%
4T1m
Isotype control
1.9%
Qa-2
94.0%
Splenocytes
Cell number
Log. Relative fluorescence

Figure S7
